# Supplementary material for: The Effect of Dietary Plant-Derived Omega 3 Fatty Acids on the Reproductive Performance and Gastrointestinal Health of Female Rabbits
Source: Vet Sci. 2024 Oct 1;11(10):457. doi: 10.3390/vetsci11100457 (PMC11512234; doi:10.3390/vetsci11100457)
Supplement: Supplementary file 1 [file vetsci-11-00457-s001.zip › vetsci-3186585-supplementary.pdf]

Supplementary material

Table S1. Feed intake among groups (CNT: receiving a commercial diet; L5%: receiving a diet modified with 5% extruded linseed; L5%PP: receiving a diet modified with 5% extruded linseed and 0.2% alga *Padina pavonica* extract) during pregnancy and lactation.

**Feed intake (g)**	**Group**									**P value**
	**CNT**			**L5%**			**L5%PP**			
	Median	Q1	Q3	Median	Q1	Q3	Median	Q1	Q3	
Pregnancy	150	150	150	150	150	150	150	150	150	0.358
Lactation	210	150	240	210	150	240	210	150	240	0.560

Values followed by the same letter in each row do not differ significantly (P<0.05).

Table S2. Crosstabulation of histological scores by group according to tissue and parameter.

Tissue	Parameter	Score	Group						P value*	Eta
			CNT		L5%		L5%PP			
			Count	N %	Count	N %	Count	N %		
Stomach	Leukocytes	0	6	100.0%	6	100.0%	6	100.0%	-	-
	Degeneration	0	6	100.0%	6	100.0%	6	100.0%	-	-
	Necrosis	0	6	100.0%	6	100.0%	6	100.0%	-	-
	Hyperplasia	0	6	100.0%	6	100.0%	6	100.0%	-	-
Duodenum	Leukocytes	0	6a	100.0%	1b	16.7%	5a	83.3%	0.012	0.764 (eta^2=0.583)
		1	0a	0.0%	5b	83.3%	1a	16.7%		
	Degeneration	0	6	100.0%	6	100.0%	6	100.0%	-	-
	Necrosis	0	6	100.0%	6	100.0%	6	100.0%	-	-
	Hyperplasia	0	6	100.0%	6	100.0%	6	100.0%	-	-
Jejunum	Leukocytes	0	2	33.3%	0	0.0%	2	33.3%	0.471	0.378
		1	4	66.7%	6	100.0%	4	66.7%		
	Degeneration	0	6	100.0%	6	100.0%	6	100.0%	-	-
	Necrosis	0	6	100.0%	6	100.0%	6	100.0%	-	-
	Hyperplasia	0	6	100.0%	6	100.0%	6	100.0%	-	-
Ileum	Leukocytes	0	0	0.0%	0	0.0%	1	16.7%	1.000	0.343
		1	6	100.0%	6	100.0%	5	83.3%		
	Degeneration	0	6	100.0%	6	100.0%	6	100.0%	-	-
	Necrosis	0	6	100.0%	6	100.0%	6	100.0%	-	-
	Hyperplasia	0	6	100.0%	6	100.0%	6	100.0%	-	-
Colon	Leukocytes	1	3a	50.0%	6b	100.0%	6b	100.0%	0.074	0.632 (eta^2=0.400)
		2	3a	50.0%	0b	0.0%	0b	0.0%		
	Degeneration	0	6	100.0%	6	100.0%	6	100.0%	-	-
	Necrosis	0	6	100.0%	6	100.0%	6	100.0%	-	-
	Hyperplasia	0	6	100.0%	6	100.0%	6	100.0%	-	-
Cecum	Leukocytes	1	6	100.0%	5	83.3%	5	83.3%	1.000	0.250
		2	0	0.0%	1	16.7%	1	16.7%		
	Degeneration	0	6	100.0%	6	100.0%	6	100.0%	-	-
	Necrosis	0	6	100.0%	6	100.0%	6	100.0%	-	-
	Hyperplasia	0	6	100.0%	6	100.0%	6	100.0%	-	-

* p value from Fisher's exact

- No computed as the score is constant

Values followed by the same subscript letter in each row do not differ ($p \leq 0.05$; z-test).

Table S3. Villi height and thickness in duodenum, jejunum, ileum, colon, and cecum. Values are medians (Md) and interquartiles (first-Q1 and third-Q3).

Intestinal tract	Parameter	Group									P value
		CNT			L5%			L5%PP			
		Md	Q1	Q3	Md	Q1	Q3	Md	Q1	Q3	
Duodenum	Height Villi	706.48	663.20	715.80	662.69	633.42	731.27	742.54	720.48	745.62	0.098
	Thickness Villi	66.82	64.33	78.76	63.82	56.63	77.29	64.26	60.35	73.91	0.630
Jejunum	Height Villi	480.16	443.30	498.92	453.35	410.58	470.53	490.10	455.86	620.47	0.158
	Thickness Villi	59.77	57.38	65.60	54.26	48.83	73.41	65.77	54.79	72.92	0.484
Ileum	Height Villi	474.77	446.64	500.66	457.18	424.80	464.91	460.13	421.44	547.75	0.751
	Thickness Villi	58.67	57.64	63.54	50.54	46.41	67.92	56.34	53.87	58.29	0.523
Colon	Thickness	251.01	233.83	285.82	291.47	266.40	302.56	254.80	243.91	282.90	0.444
Cecum	Thickness	134.19	108.24	147.67	123.78	121.34	126.89	137.24	121.74	144.90	0.778

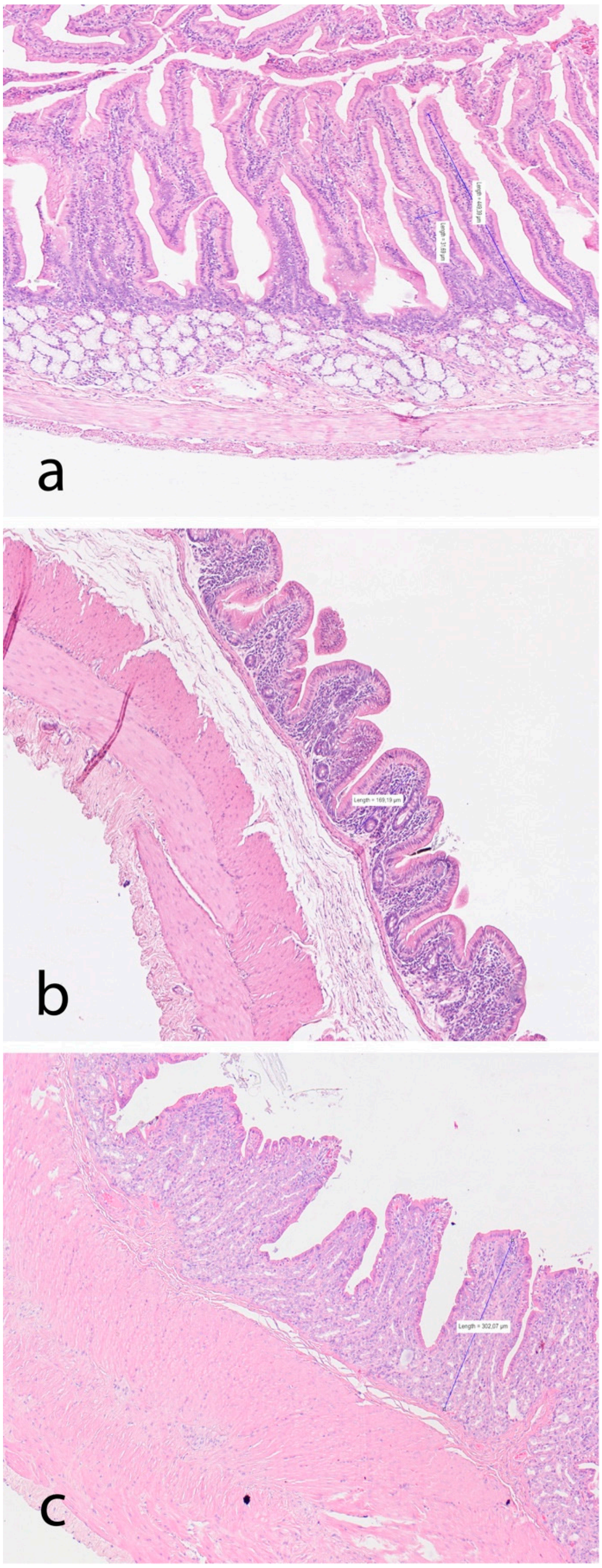

Figure S1. Gastrointestinal tract. Histological evaluations. (**a**) Measurement of villus height (from the apex to the base) and thickness (from one lateral extremity to the other) in the duodenum (x100). Measurement of cecum (**b**) and colon (**c**) thickness from the luminal aspect of the lamina propria to the muscularis mucosae (x400).
